# Influence of Air Temperature and Humidity on Dehydration Equilibria and Kinetics of Theophylline

**DOI:** 10.1155/2013/892632

**Published:** 2012-12-05

**Authors:** Amira Touil, Roman Peczalski, Souad Timoumi, Fethi Zagrouba

**Affiliations:** ^1^Ecole Nationale d'Ingénieurs de Gabès (ENIG), Université de Gabès, rue Omar Ibn-Elkhattab, Gabès 6029, Tunisia; ^2^Université de Lyon, Université Lyon 1, 69622 CNRS, UMR 5007, Laboratoire d'Automatique et Génie des Procédés (LAGEP), Campus de la Doua, bât. CPE, 3 rue Victor Grignard, 69616 Villeurbanne, France; ^3^Institut Supérieur des Sciences Appliquées et de Technologie de Gabès (ISSAT), Université de Gabès, rue Omar Ibn El-Khatab, Gabès 6029, Tunisia; ^4^Institut Supérieur des Sciences et Technologies de l'Environnement (ISSTE), Université 7 Novembre de Carthage, Technopôle de Borj Cédria, Hammam-Lif 1003, Tunisia

## Abstract

The effect of hygrothermal conditions (air temperature and relative humidity) on the dehydration of theophylline monohydrate was investigated. Firstly, the equilibrium states of theophylline were investigated. The data from gravimetric analysis at constant temperature and humidity were reported as desorption isotherms. The PXRD analysis was used to identify the different polymorphic forms of theophylline: the monohydrate, the metastable anhydrate, and the stable anhydrate. Solid-solid phase diagrams for two processing times were proposed. Secondly, the dehydration kinetics were studied. The water content evolutions with time were recorded at several temperatures from 20°C to 80°C and several relative humidities from 4% to 50%. Different mathematical models were used to fit the experimental data. The spatially averaged solution of 2D Fickian transient diffusion equation best represented the water mass loss versus time experimental relationship. The dehydration rate constant was found to increase exponentially with air temperature and to decrease exponentially with air relative humidity.

## 1. Introduction

Theophylline (C_7_H_8_N_4_O_2_: 3,7-dihydro-1,3-dimethyl-1H-purine-2,6-dione) is widely used as a bronchodilator in asthma therapy. Its hydration behaviour and solid-state transitions have been extensively studied and presently the compound is known to exist as a monohydrate (M) and four anhydrous polymorphs (I, II, III, and IV) [1–3].

The M form has been shown to lose water in low relative humidity (or water activity for solvents) to produce form II which is the most prevalent form and has been long considered as the only stable form at room temperature [4, 5]. Form I is produced by heating form II [2, 6] and is reported to be the stable form at higher temperatures. Form III is a highly metastable form and converts easily to form II during storage [3, 7]. This form can be only produced as an intermediate during dehydration of the monohydrate and was never found as an intermediate during hydration of the stable anhydrous form according to Phadnis and Suryanarayanan [8]. Form IV has been identified recently [9]. It occurs as a result of slow, solvent-mediated conversion from form II, and is now claimed as the most thermodynamically stable anhydrous polymorph of theophylline. However, in subsequent sections of the paper the term “stable anhydrate” will refer only to form II as this form is the most commonly encountered and discussed in the literature and the one which was obtained in the dehydration experiments reported here.

The dehydration kinetics of theophylline monohydrate has been the subject of several investigations. Airaksinen et al. [11] investigated the effect of two drying methods on the content of different theophylline polymorphs (form II, form III, and form M) in the final product. They reported that the metastable polymorph (III) was an intermediate form during the transition from monohydrate to anhydrate form II and that the relative amounts of the coexisting polymorphs were affected by humidity and temperature. Metastable anhydrous theophylline (III) was the major form that was produced at temperatures of 40–50°C with both MMFD (multichamber microscale fluid bed dryer) and VT-XRPD (variable temperature X-ray powder diffractometer) drying apparatus. Drying at temperatures over 50°C with the VT-XRPD produced mostly stable anhydrous theophylline, but over 20% metastable anhydrous theophylline remained even at 90°C. Drying in an MMFD produced only stable anhydrous theophylline at 60°C. 

Nunes et al. [12] investigated the dehydration of theophylline monohydrate using the 2D XRD technique. The analysis of the diffraction data revealed that the dehydration of the monohydrate occurred by two pathways, the first involving the formation of the metastable anhydrous III polymorph. The dehydration started with the transition from monohydrate to metastable anhydrate, but after a delay was continued by transition from monohydrate to stable anhydrate. The rate constants for individual transition steps were determined by fitting the data to solid-state reaction models. The two steps were best described by a first-order rate equation and by the Avrami-Erofeev's equation with the power factor *n* of 0.33, respectively. Lower temperatures resulted in higher concentration of form III in the dehydrated product.

Suzuki et al. [2] reported that the dehydration under isothermal conditions obeyed the Avrami-Erofeev's equation with power factor n of 0.5. Duddu et al. [13] reported that the dehydration of theophylline at 47°C was a single-step process and could be best described by an Avrami-Erofeev's equation with *n* = 0.25 and that at 40°C the dehydration of theophylline was a two-step process. Each step could be linearized using the Avrami-Erofeev's relationship with *n* = 0.25. The same conclusion was given by Agbada and York [14].

Matsuo and Matsuoka [3] have found that relative humidity and temperature are major controlling factors for the solid-state polymorphic transitions of theophylline anhydrate from the metastable phase (form III) to the stable phase (form II). They proposed a two-step serial kinetic model consisting of a chemical first-order rate equation and a Fickian diffusion-based rate equation. They suggested also a simple method to correlate the rate constants as functions of temperature and humidity. Their approach was extended to the transition from theophylline monohydrate to stable anhydrate in our study.

It follows from the above short literature review, that the dehydration rates of theophylline were studied mainly with temperature as the controlling parameter and the influence of relative humidity has not been much investigated. The aim of this work was to experimentally study the influence of both parameters, varying in a broad range, on the solid phase transition pathways and kinetics during dehydration of theophylline. From equilibrium data, a phase diagram was determined and from kinetic data a simple predictive model for dehydration progress with time was derived. The model proposed here was compared to the common ones from the literature concerning the dehydration of theophylline and was proven to interpret better the experimental curves. Unlike the others, this model included explicitly the relative humidity and was based on water diffusion as rate controlling transition mechanism.

## 2. Materials and Methods

### 2.1. Materials

Anhydrous theophylline was purchased from Sigma-Aldrich (St. Louis, MO, USA). Hydrated theophylline was recrystallised from a saturated solution of anhydrous theophylline. Anhydrous theophylline was first dissolved in hot (60°C) distilled water. The solution was then filtered and allowed to cool overnight. Hydrated theophylline crystals were harvested and dried at room temperature. CsF, LiCl, MgCl_2_. 6H_2_O, Mg(NO_3_)_2_, K_2_CO_3_, NaBr, NaCl and KCl salts used for hygrothermal conditioning of hydrated theophylline samples were also supplied by Sigma-Aldrich.

### 2.2. Differential Scanning Calorimetry (DSC)

A differential scanning calorimeter (Model Q200, Thermal Analysis Instruments) was used. The instrument was calibrated with pure indium. About 4-5 mg theophylline sample was packed in aluminum pans, crimped with lids having several pinholes. Tests were run from room temperature to 300°C, at a heating rate of 5°C/min under dry nitrogen purge.

### 2.3. Thermogravimetric Analysis (TGA)

The thermogravimetric analyser (TGA Netzsch TG 209 F1 Iris ASC) was used. The sample was heated in an open aluminum pan from room temperature to 200°C, under nitrogen purge, at a rate of 1°C/min.

### 2.4. X-Ray Diffractometry

X-Ray powder (XRPD) theta-theta diffractometer (Bruker axs D8, Bruker AXC GmbH, Karlsruhe, Germany) was used. The XRPD experiments were performed in symmetrical reflection mode with CuK_*α*_ radiation (*λ* = 1.54 Å) using Göbel mirror bent gradient multilayer optics. The scattered intensities were measured with a scintillation counter. The angular range was 5–50° with a scan speed of 5° 2*θ* min^−1^.

### 2.5. Isothermal Gravimetric Method

Saturated solutions of CsF, LiCl, MgCl_2_·6H_2_O, Mg(NO_3_)_2_, K_2_CO_3_, NaBr, NaCl, and KCl were prepared and stored at 10, 20, 30, 40, 50, and 80°C providing the different required relative humidity conditions. The relative humidity values were verified with an Activity-meter (GBX Scientific Instruments, FA-st/1, France) and are detailed in Table 1. The samples of theophylline monohydrate were placed in closed containers filled with the solutions. Samples were weighed periodically until constant weight was obtained.

### 2.6. Scanning Electron Microscopy (SEM)

Hitachi S-800 Field Emission Gun (FEG) Scanning Electron Microscope (SEM) was used. All samples were coated with gold for increased signal/noise ratio.

### 2.7. Dehydration Rate Models

In order to interpret and fit the experimental dehydration curves, several equations were proposed in the literature. Among them, the models A and B are the most widely used for solid-state transitions (see Table 2). Concerning the Avrami-Erofeev's model, the power factor of 0.5 corresponded best to our experimental data. Moreover, our goal was to verify also a different model where the dehydration will be considered like a diffusion driven drying process. This model, labelled C in Table 2, was derived from the Fick's transient diffusion equation by spatially averaging a 2D solution for an infinite square section rod and by limiting the infinite series solution to the first term [10]. The square section rod was chosen as the simplified geometrical representation of the theophylline monohydrate crystals' shape.

## 3. Results and Discussion

### 3.1. Identification of Starting Materials

**Figure 1:**
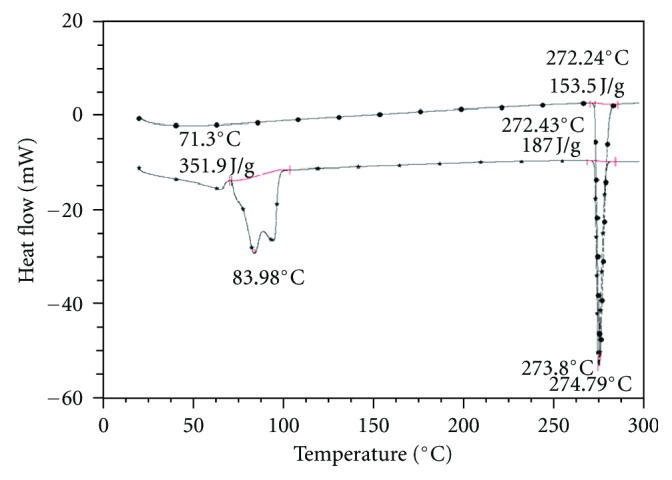
DSC record for commercial (–*∙*–) and recrystallized theophylline (–**∗**–).

**Figure 2:**
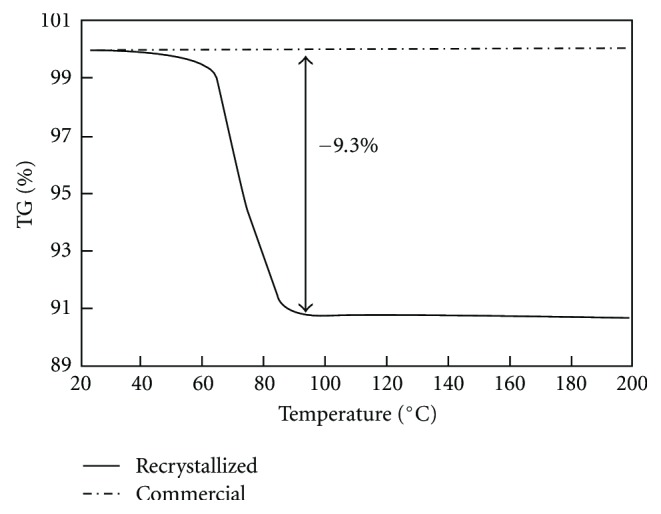
TGA record for commercial and recrystallized theophylline.

DSC and TGA curves for the recrystallized and commercial theophylline are shown in Figures 1 and 2. The DSC curve of the recrystallized product showed a broad two-split endothermic peak around 60–90°C and a sharp endothermic peak at 272°C. The first endothermic peak was due to dehydration and corresponded to the weight loss of 9.3% in the range from 60 to 80°C on the TGA curve. The stoichiometric weight loss value (calculated for one mol of water) was almost identical to the value obtained from the TG analysis. Thus, the recrystallized product corresponded effectively to theophylline monohydrate. The second endothermic peak was due to the melting of the anhydrous form. The broadness and split of the dehydration peak were possibly due to the broadness and bimodal character of the hydrated form crystal size distribution (available but not presented here). The dehydration was perhaps initiated with smaller crystals and ended with bigger ones as the kinetics constants could be dependent on the particle size.

The DSC curve of commercial theophylline showed only one endothermic peak at 272°C due to melting and no weight loss was observed on the TGA curve. Therefore, the commercial product appeared effectively as the anhydrous form of theophylline. Similar results were reported by Suzuki et al. [2] and Ono et al. [15].

**Figure 3:**
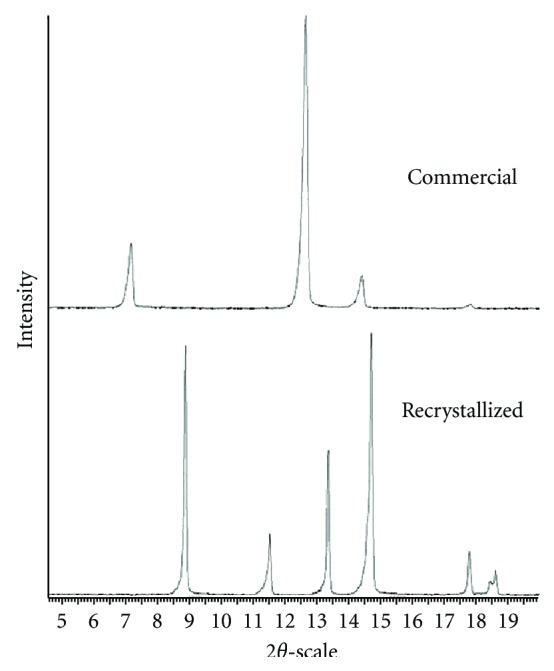
PXRD patterns of theophylline.

The powder X-ray diffraction patterns of the recrystallized and commercial theophylline are shown in Figure 3. These patterns were in excellent agreement with those reported in the Powder Diffraction File (International Centre for Diffraction Data) [16] and by Suzuki et al. [2] for the monohydrate and anhydrate stable form II, respectively. 

### 3.2. Desorption Isotherms and Solid-Solid Phase Diagram of Theophylline

**Figure 4:**
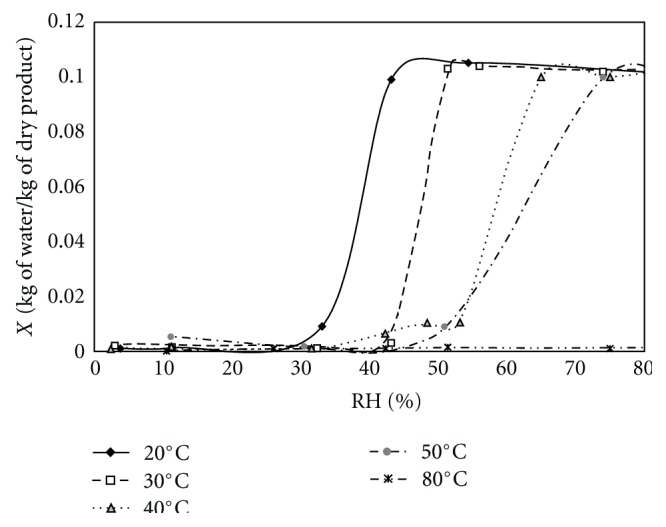
Desorption isotherms of theophylline.

The final equilibrium contents obtained from gravimetric experiments at constant temperature and constant relative humidity were used to plot the desorption isotherms of theophylline. The plot of desorption equilibriums (dry basis water content *X* versus air relative humidity RH) in Figure 4 presents one plateau for high humidities which corresponds to the hydrated form and a plateau for low humidities which correspond to the anhydrous form.

The hygroscopic threshold was found at moisture content *X* = 0.1 which is stoichiometrically equivalent to the monohydrate. These results showed the monohydrate to be present at relative humidities higher than 43.2%, 51.4%, 65%, and 74% at temperatures of 20°C, 30°C, 40°C, and 50°C, respectively. The increasing relative humidity required to form a hydrate at higher temperatures can be explained by the weakening of H-bonds between the water and theophylline [17].

PXRD analysis confirmed that the 10% water content phase (see Figure 4), was the theophylline monohydrate and that the zero water content phase corresponded to the anhydrate, form II or III. The diffractogram of the form III is not referenced in the Powder Diffraction File of ICDD but is presented in a paper by Nunes et al. [12]. By means of the PXRD analysis, two diagrams showing the occurrence of the different forms of theophylline as a function of hygrothermal conditions were determined.

**Figure 5:**
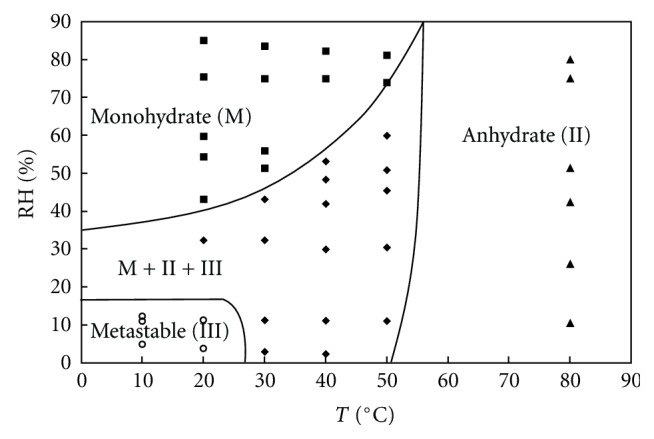
Solid-solid state diagram of theophylline after 12 hours of processing at different environmental conditions.

The first diagram (see Figure 5) presents the solid states for theophylline processed at different hygrothermal conditions (relative humidities and temperatures) during 12 hours. Strictly speaking, this diagram which represents transient and non equilibrium states of the product should not be called a phase diagram.

This diagram shows the domains of existence of the monohydrate, the stable anhydrate and the metastable anhydrate, after 12 hours. The metastable domains corresponded to temperature ranging from 0 to around 27°C and relative humidity ranging from 0% to 20%. For temperatures higher than 50°C, the anhydrate form II was the only one to occur at all relative humidities. The monohydrate form dominated at high relative humidities (higher than 40%) and low temperatures. The three forms of theophylline coexisted for moderate temperatures and humidities.

**Figure 6:**
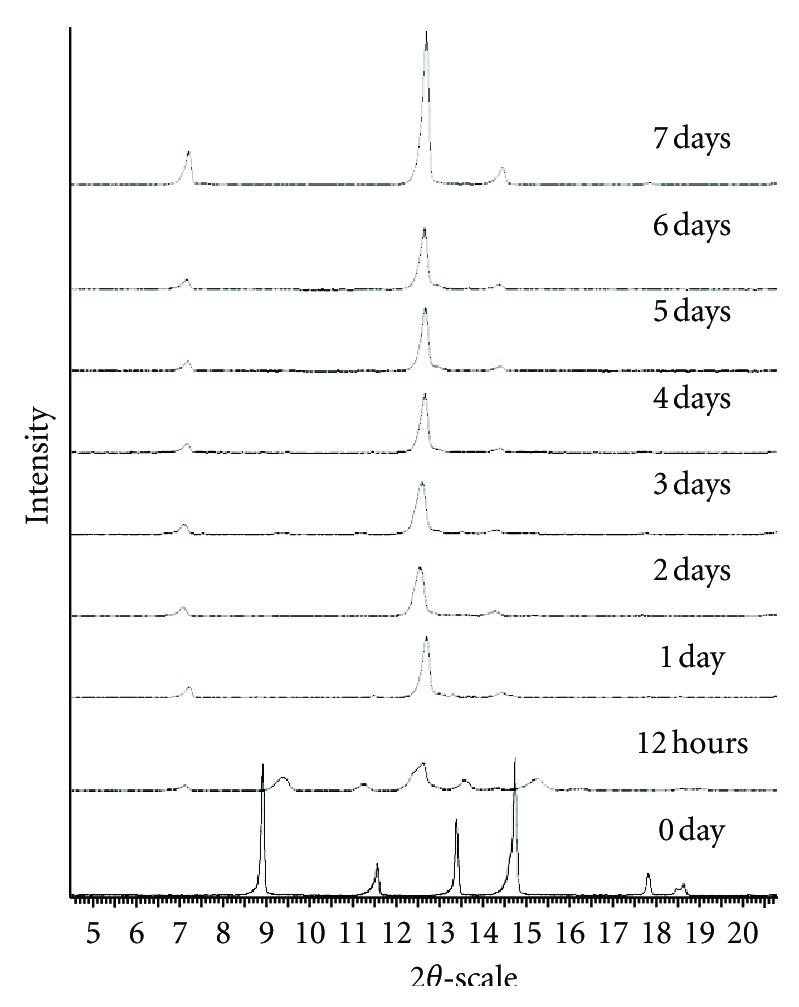
PXRD patterns of theophylline processed at 3.8% RH and 20°C.

The evolution of PXRD patterns of theophylline during processing at 3.8% and 20°C (conditions which favor the occurrence of the anhydrous metastable form) is presented in Figure 6. The pattern after 12 hours corresponds to a mixture of the monohydrate M, the metastable anhydrate III [12], and stable anhydrate II but with a prevalence of form III. One can observe in this pattern that the most noticeable characteristic peaks are those of the form III (9.4, 11.3, 12.5, and 15.4°2*θ*).

**Figure 7:**
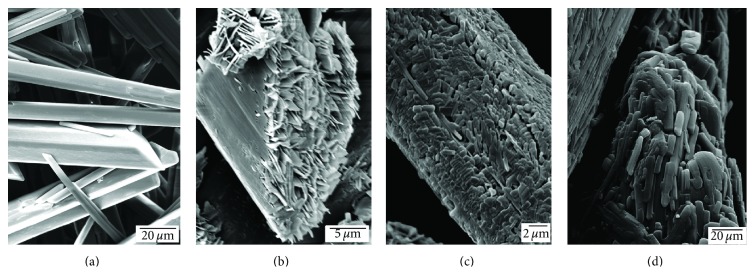
Scanning electron micrographs of different phases of theophylline at 3.8% RH and 20°C. (a) 0 hours (monohydrate), (b) after 12 hours (metastable anhydrate), (c) after 24 hours (stable anhydrate), and (d) after 7 days (stable anhydrate).

The transition between the monohydrate and the metastable form corresponds to the reversible loss of water through channels in the crystal lattice according to Phadnis and Suryanarayanan [8]. The crystal lattice of the metastable form was observed to collapse with time to produce the anhydrous stable form (see Figure 7).

**Figure 8:**
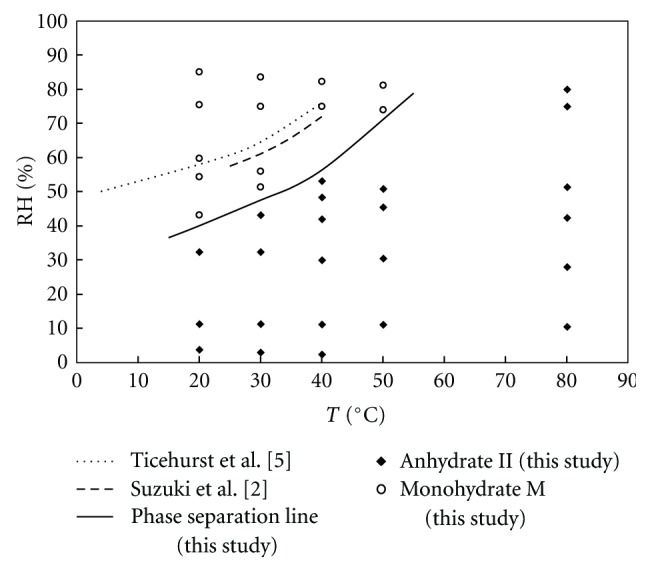
Solid-solid phase diagram of theophylline after one month of processing at different environmental conditions.

A second solid-solid phase diagram corresponding to equilibrium states (after one month) of theophylline monohydrate processing at different relative humidities and different temperatures is presented on Figure 8. It exhibits only the two forms of theophylline: the monohydrate (M) and the stable anhydrate form (II). The data from studies of Suzuki et al. [2] and Ticehurst et al. [5] are also shown.

Our results did not agree closely with the above cited literature data. Ticehurst et al. [5] used acetone/water slurry bridging experiments to study the phase conversion of theophylline. Their results showed that theophylline monohydrate was present for RH > 50% at 4°C, for RH > 64% at 30°C, and for RH > 76% at 40°C. The reason for the discrepancy between our data and those of Ticehurst et al. could be the solubility of the solute in the solvent mixture. Suzuki et al. [2] used the BET analysis for the determination of the vapor pressure (proportional to RH) at different temperatures. The difference between their data and ours could be due to the shorter time of their experiments. It could be supposed that this time was not long enough to reach the definitive thermodynamic equilibrium. In our case, one month can be considered long enough.

### 3.3. Dehydration Curves of Theophylline

**Figure 9:**
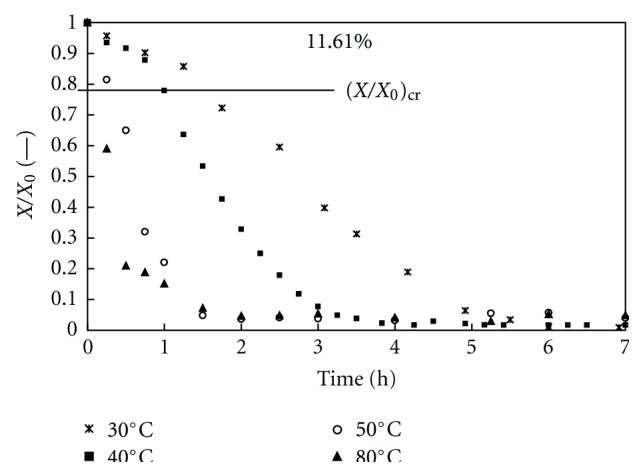
Representative theophylline monohydrate's dehydration profilesat 11.61% RH and different temperatures.

The water content *X* (divided by its initial value *X*_0_) versus time plots for the dehydration of theophylline monohydrate at 11.61% relative humidity and at different temperatures (30°C, 40°C, 50°C, and 80°C) is shown in Figure 9. The dehydration rate increased strongly with temperature. Similar results were reported by Suzuki et al. [2], Agbada and York [14], and Nunes et al. [12].

**Figure 10:**
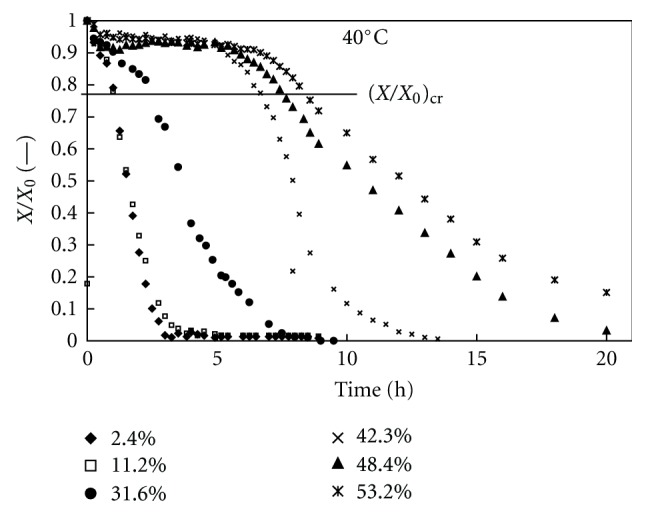
Theophylline monohydrate's dehydration profiles at 40°C and different relative humidities.

The water content *X* (divided by its initial value *X*_0_) versus time plots for the dehydration of theophylline monohydrate at 40°C, and at different relative humidities (2.4%, 10.5%, 31.6%, 42.3%, 48.4%, and 53.2%) is presented in Figure 10. The dehydration rate decreased considerably with relative humidity.

These experiments showed that both temperature and humidity affected the dehydration rate of theophylline monohydrate. The same kinds of curves were also obtained for all temperatures and relative humidities considered, but these results are not shown here.

Most of the experimental curves exhibited two dehydration stages: the first stage (1 ≤ *X*/*X*_0_ ≤ 0.8) with a slow quasilinear trend and a second stage (0.8 ≤ *X*/*X*_0_ ≤ 0) with a fast quasiexponential trend. The first stage was practically neither observed for the highest applied temperatures when probably form II was exclusively produced nor for the lowest relative humidity when probably the form III was exclusively produced. According to the literature [11–14], the first stage could correspond to a predominant transition to form III and the second stage to a predominant transition to form II with different mechanisms and rates of dehydration. If the classification proposed by Galwey [18] is adopted, the mechanism of the former transition would be “WET3” with progressive crystal reorganization because of water departure from an interface advancing at a constant rate. The mechanism of the latter transition would be “WET4” with initial crystal destruction and recrystallization driven by water departure by diffusion at a falling rate. 

### 3.4. Modeling of the Second Stage of Dehydration of Theophylline

**Figure 11:**
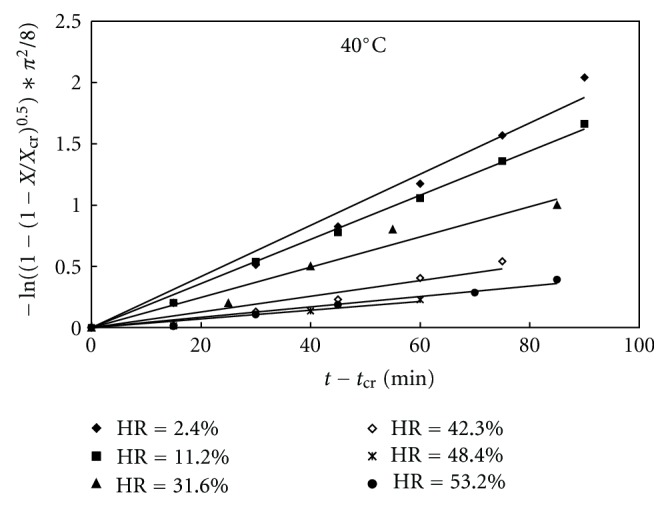
Dehydration data of theophylline monohydrate fitted by model C at 40°C and different relative humidities.

In the following quantitative analysis only the second stage will be considered in the limits from 0.8 to 0.1 of the relative water content. The models A, B, and C introduced in Section 2.7 were fitted to our experimental data. First, the data were plotted according to the linearized equations given in Table 2. The experimental points and fitting lines for model C for dehydration at 40°C at different relative humidities are shown in Figure 11. From the slopes of the fitted straight lines, the values of dehydration rate parameter *k* were evaluated. The *k* values for the different models and the corresponding correlation factor *R* ^2^ are given in Table 3.

The best fit over all experimental conditions was obtained with the model C. The *k* parameter was considered as a function of air temperature and relative humidity and was represented by the classical Arrhenius equation:

(1) lnk=lnk0−EaRT.

**Figure 12:**
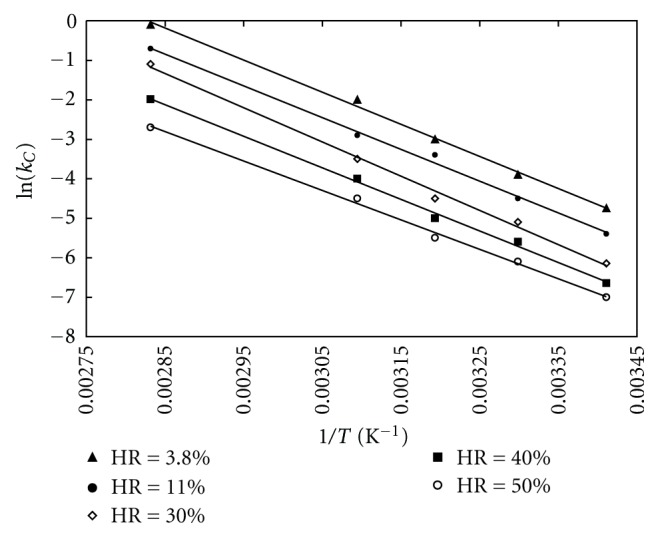
Logarithm of rate constant as function of temperature fitted by Arrhenius equation for dehydration of theophylline monohydrate at different relative humidities.

**Figure 13:**
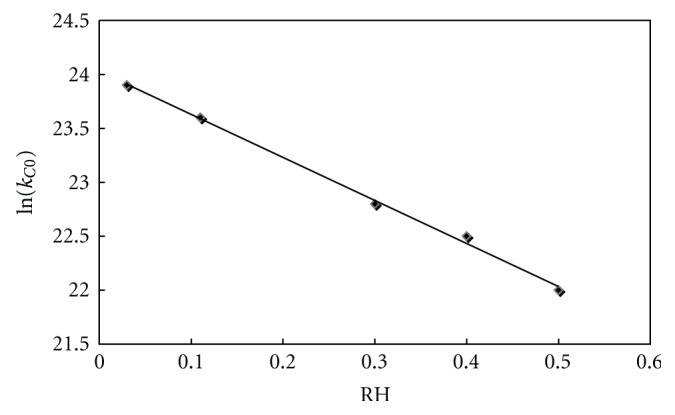
Logarithm of preexponential factor in Arrhenius equation versus relative humidity for the dehydration of theophylline monohydrate.

**Figure 14:**
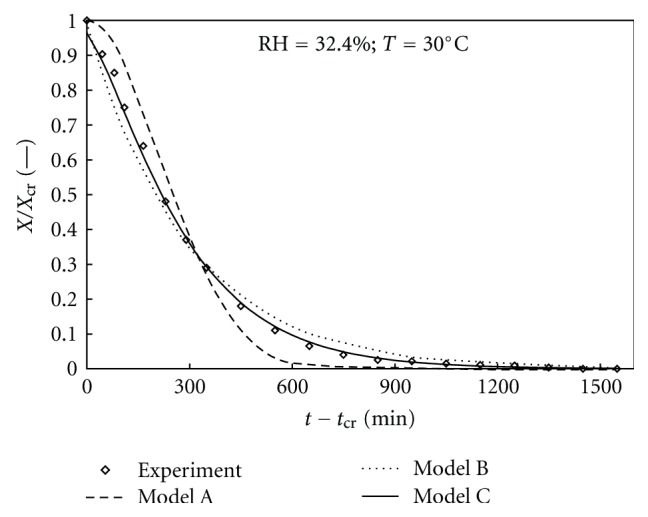
Predicted dehydration curves of theophylline monohydrate according to different models.

According to the above equation, ln*  k* was plotted as function of the inverse of the temperature. As the relative humidity imposed by salt solutions (see Table 1) varies slightly with temperature, the relative humidity values given in Figure 12 and Table 4 were averaged over the considered temperature domain (20–80°C). From the linear fits (see Figure 12), the activation energy *E*_*a*_ (values given in Table 4) and the preexponential coefficient *k*_0_ (values given in Figure 13) were calculated as function of relative humidity. These values were close to those calculated by Suzuki et al. [2] and Agbada and York [14]. The activation energy appeared to be varying very little with relative humidity and was approximated by its average value (see Tables 4 and 5). On the contrary, the logarithm of the preexponential factor *k*_0_ was found to decrease linearly with the relative humidity (see Figure 14).

The fitting coefficients *m* and *n* of the expression

(2) lnk0=mRH+n

are given in Table 5. The best correlation factors *R* ^2^ were again obtained with the model C.

**Figure 15:**
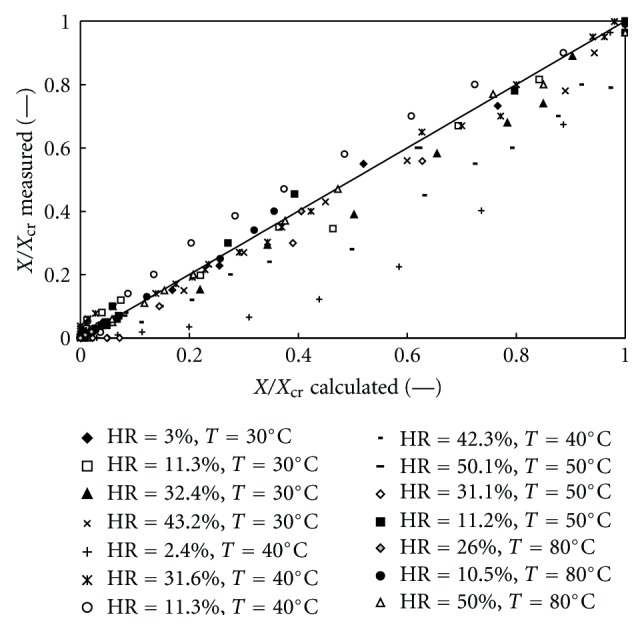
Experimental versus predicted data (model C) for the dehydration of the theophylline monohydrate.

Thus, the Fickian diffusion-based model (C) coupled with the Arrhenius expression for *k* (with constant activation energy *E*_*a*_) and a linear expression for ln*k*_0_ could be a possible mathematical representation of the theophylline's dehydration curves during the second stage. This representation would include the influence of both temperature and relative humidity in a quite simple way. The predicted dehydration curves of the theophylline monohydrate, according to different models are shown in Figure 14 for one particular operating condition. In order to check the global validity of this approach, the water content values corresponding to experimental recording times were calculated according to (∗∗∗), (1), and (2) with the parameters values given in Table 2. Then, the calculated water content values were plotted versus the experimental ones in Figure 15 for all considered operating conditions.

Except for the lowest relative humidity (2.4%) and the highest temperature (80°C), the points are rather narrowly distributed along the bisecting line and thus the correlation between the two sets of data can be considered satisfactorily.

The capability of the model C to interpret correctly the experimental data may indicate that the dehydration rate of theophylline in the second (final) stage of the process is controlled by water transfer within the crystal rather than the nucleation and growth of the new crystal phase or the progression of the interface between the new and old phases. This corroborates the hypothesis of a “WET4” dehydration mechanism with a process rate controlled by diffusion suggested at the end of Section 3.3.

Theophylline is classified as a lattice channel (or tunnel) hydrate, which class is known for releasing water rather easily. However, unlike in many others channel hydrates, theophylline's molecules are very tightly packed and its channels are particularly narrow (roughly of the size of the water molecule) according to Ahlqvist and Taylor [19]. Water mobility is then very reduced and the mass transfer limitation will be particularly strong in such a case.

## 4. Conclusions

The effect of hygrothermal conditions (air temperature and relative humidity) on the dehydration of theophylline monohydrate was investigated. The results of gravimetric experiments provided the conditions where the monohydrate transforms to the anhydrate. The analysis of PXRD patterns allowed the determination of the conditions where only one of the anhydrate forms was obtained or where the two forms could coexist. The metastable form appeared preferably for low temperatures and low humidities, while the stable form was preferred for high temperatures. The coexistence occurred only for short processing times (few days). The proposed solid-solid phase diagram can be very useful for predicting the state of theophylline during drying or during storage.

The dehydration rate was found to increase strongly with air temperature and to decrease with air relative humidity. Three mathematical models: Avrami-Erofeev's solid-state transition, first order chemical kinetic, and Fick's 2-D diffusion were used to fit the experimental data. The measured evolutions of water mass with time during dehydration of theophylline for all considered conditions were found to be best represented by the spatially averaged solution of 2-D Fickian transient diffusion equation. The parameters of this equation were correlated to temperature and relative humidity by exponential expressions, and finally a simple but comprehensive model for prediction of the dehydration progress with time was obtained. This kind of procedure of fitting semi-empirical equations to dehydration curves can provide a fast way for drying process modeling and optimal control in pharmaceutical industry.

## Figures and Tables

**Table 1:** Temperature and relative humidity conditions applied for the dehydration experiments.

Salt	Temperature (°C) and relative humidity (%)					
	10°C	20°C	30°C	40°C	50°C	80°C
CsF	4.9	3.8	3.0	2.4	2.1	2.6
LiCl	11.3	11.3	11.3	11.2	11.1	10.5
MgCl_2_	—	33.1	32.4	31.6	30.5	26.1
K_2_CO_3_	—	43.2	43.2	42.3	45.6	42.4
Mg(NO_3_)_2_	—	54.4	51.4	48.4	45.4	45.0
NaBr	—	59.1	56.0	53.2	50.9	50.9
NaCl	—	75.5	75.1	74.7	74.4	74.4
KCl	—	85.1	83.6	82.3	81.2	81.2

**Table 2:** Dehydration rate models.

Model	Description
(A) (−ln(*X*/*X*_cr_))^1/2^ = *k*(*t* − *t*_cr_) (∗)	Avrami-Erofeev's equation for solid state transition by random nucleation [2].
(B) −ln(*X*/*X*_cr_) = *k*(*t* − *t*_cr_) (∗∗)	classical first order chemical kinetics [2].
(C) −ln((1 − (1−*X*/*X*_cr_)^1/2^)*π* ^2^/8) = *k*(*t* − *t*_cr_) (∗∗∗)	spatially averaged solution of 2-D Fick's transient diffusion equation [10].

*X*_cr_ = 0.8*X*_0_ is the fitting start water content, *X*_0_ is the product initial water content, and *t*_cr_ is the fitting start time.

**Table 3:** Values of the rate constants for the three models (*k*_*A*_, *k*_*B*_, and *k*_*C*_).

*T*°C	Humidity (%RH)	*k*_*A*_ (min^−1^) × 10^−3^	*R* ^2^ (—)	*k*_*B*_ (min^−1^) × 10^−3^	*R* ^2^ (—)	*k*_*C*_ (min^−1^) × 10^−3^	*R* ^2^ (—)
	3.8	1.0	0.885	0.8	0.998	1.2	0.999
20	11.6	0.4	0.985	0.1	0.934	0.3	0.992
	32.0	9.4	0.927	1.0	0.927	8.1	0.928

	3.0	10.4	0.95	12.1	0.954	15.8	0.979
	11.3	7.5	0.967	7.7	0.950	10.4	0.977
30	32.0	3.1	0.962	1.0	0.976	1.5	0.996
	43.0	39.4	0.935	59.8	0.999	71.7	0.999
	51.0	8.6	0.831	5.9	0.753	9.0	0.899

	2.4	4.9	0.951	2.2	0.852	3.7	0.869
	11.2	3.3	0.866	12.5	0.988	18.7	0.999
40	31.6	12.7	0.921	14.6	0.992	19.1	0.979
	42.3	5.2	0.7815	2.8	0.991	4.4	0.993
	48.4	7.4	0.9318	4.4	0.981	7.9	0.985
	53.2	8.0	0.711	2.2	0.985	3.6	0.995

	10.6	5.6	0.937	2.6	0.991	4.4	0.996
50	30.0	27.8	0.830	28.6	0.989	38.7	0.969
	50.0	16.2	0.941	19.4	0.979	2.5	0.986

	10.1	5.8	0.904	10.9	0.938	9.2	0.897
	26.0	23.8	0.962	30.6	0.973	38.6	0.996
80	44.0	41.5	0.986	82.5	0.968	98.0	0.979
	51.4	2.9	0.806	21.2	0.954	32.0	0.946

**Table 4:** Activation energies for each model equation.

Humidity (%RH)	*E*_*a*_ (kJ/mol)					
	Model A	*R* ^2^ (—)	Model B	*R* ^2^ (—)	Model C	*R* ^2^ (—)
3.8	67.9	0.989	68.7	0.993	66.2	0.997
11	72.5	0.996	72.9	0.997	65.5	0.995
30	69.3	0.994	76.6	0.998	70.5	0.996
40	72.0	0.994	72.8	0.990	65.1	0.997
50	70.8	0.993	76.0	0.991	60.6	0.996

**Table 5:** Values of constant parameters of each model equation.

	*E*_*a*_ (kJ/mol)	*m* (—)	*n* (—)	*R* ^2^ (—)
Model A	70.5	−5.26	24.18	0.978
Model B	73.4	−6.37	26.09	0.983
Model C	65.58	−3.82	22.65	0.989
